# Lectures on Diseases of the Heart

**Published:** 1888-04

**Authors:** 


**Lectures on Diseases of the Heart.** By ALONZO CLARK, M. D., LL.D., Emeritus Professor of the Principles and Practice of Medicine, College of Physicians and Surgeons, etc. New York: E. B. Treat & Co. 1887.

This work of the late Dr. Clark embodies the more important of the clinical lectures which, for many years, he gave in the hospital amphitheatre and lecture-room. Hundreds of his former pupils will thank the publishers for putting them in a permanent form. Few teachers have been endowed with so clear and concise a style of delivery. In discussing the pathology and diagnosis of cardiac lesions, the author is particularly clear. We consider this work, the result of half a century of clinical experience and study, to be one of the most valuable contributions to the literature of cardiac diseases ever produced in America.

